# Neuromuscular conditioning strategies and their effectiveness in preventing injuries in soccer players: a systematic review

**DOI:** 10.3389/fspor.2026.1930100

**Published:** 2026-09-30

**Authors:** César Eubelio Figueroa-Pico, Silvia María Castillo-Morocho, Noemí Georgina Díaz-Meneses, Andrea Patricia Chancay-Mendoza, Juan Carlos Suárez-Fernández, Juan Carlos Briones-Olvera, Jorge Eduardo Bejarano-Macías, Emilio Sebastián Portés-Montalvo, Diego Andrés Portés-Montalvo, María Fernanda Calderón León, Raúl Antonio Burgos-Macías, Javier Aquiles Hidalgo-Acosta

**Affiliations:** 1 University of Murcia, Murcia, Spain; 2 Bolivarian University of Ecuador, Guayaquil, Ecuador; 3 Technical University of Manabí, Portoviejo, Ecuador; 4 Technical University of Manabí-Pontifical Catholic University of Ecuador, Portoviejo, Ecuador; 5 Catholic University of Santiago of Guayaquil, Guayaquil, Ecuador; 6 University of Guayaquil, Guayaquil, Ecuador

**Keywords:** athletic injuries, physical activity-related injuries, soccer, sports injuries, training

## Abstract

**Background:**

The objective of this review is to define the advances in neuromuscular training and its effectiveness in preventing injuries in soccer.

**Methods:**

Eligibility criteria were randomized clinical trials on the research topic. To evaluate the number of available studies, a population comprised of soccer players who performed physical training using neuromuscular control and its effect on injury prevention was established.

The articles were selected according to the 2020 PRISMA declaration, obtained from databases such as PubMed, MEDLINE, Embase, CENTRAL, and Web of Science. For the research design, a research question was posed to define the search: What are the advances in neuromuscular training and its effectiveness in preventing injuries in soccer?

Risk of bias of individual studies was measured using the RoB2 risk of bias tool.

The results were summarized in Excel spreadsheets based on the population and interventions performed, and a descriptive analysis of the results obtained regarding training progress and its role in injury prevention was performed.

**Results:**

The studies included in the selection were those focused on reducing injuries in soccer; a total of 15 randomized controlled trials met the inclusion criteria. Three studies were excluded because they did not focus on reducing injuries, although they did improve skills and knowledge.

This study focuses on the importance of establishing injury prevention strategies in soccer, considering the different types and varieties of physical training currently used due to the high injury rate. Strength and balance training reduced ankle instability and kinesiophobia (*P* < 0.001). The additional post-FIFA 11+ exercise program significantly reduced the total number of injuries (*P* = 0.001) and the initial injury incidence (*P* = 0.003) compared to the pre-FIFA 11+ training program. Lower extremity injuries for the M11+, F11+, and control groups were 2.00, 5.79, and 7.55 per 1,000 hours of training, respectively, with M11+ having the lowest injury incidence (*P* = 0.02). A significant reduction in the risk of anterior cruciate ligament (ACL) injury was observed after the FIFA 11+ program compared to the pre-program rate (*P* = 0.031). The extended knee control training group had a 29% lower incidence of injuries than the comparison group (*P* = 0.083) (incidence ratio 0.71; 95% CI 0.52 to 0.98). The extended knee control program included a running warm-up and six exercises focused on muscle strength and neuromuscular control, with 10 variations.

**Discussion:**

Limitations of the evidence show poor program adherence and limited research on the causes of dropouts and their relationship to injuries. Data are based on heterogeneous populations to integrate results on a large scale; However, more high-quality research in specific populations is needed.

Interpretation of the role of physical training in injury prevention yielded significantly high results, highlighting the importance of its implementation. Factors that have a negative effect on injuries, such as training discontinuation, were also observed.

**Systematic Review Registration:**

identifier 1140426.

## Introduction

### Justification

The justification for this research focuses on the importance of establishing best practice strategies for preventing injuries in soccer with the different types and varieties of physical training currently used due to the high injury rate.

Sports medicine is the intersection of physical education and medicine. This branch of medicine focuses on the physiological, anatomical, and educational outcomes, as well as the management of sports-related pathologies. In soccer, an exercise program allows for proper neuromuscular control, establishing more efficient communication between muscles and the central nervous system, thus improving mobility.

Sports medicine deals with the prevention, diagnosis, and treatment of injuries related to physical activity. This requires a group of interventions that include physical education, neuromuscular training, educational sessions, biofeedback, and landing mechanics training. It also recognizes other biomechanical risk factors that can affect the practice of soccer.

Neuromuscular training, multicomponent neuromuscular warm-up programs, strength-focused interventions, balance training, proprioception, rehabilitation, educational participation, and technology-assisted or biofeedback interventions allow for improved neuromuscular control in players. These techniques promote communication between muscles and the central nervous system. In the field of soccer, these types of practices are applied to improve neuromuscular control and prevent injuries.

Neuromuscular control refers to the coordination of muscles that allows for joint stability. Adequate training improves neuromuscular control; Otherwise, it is a risk factor for knee and other injuries. Lack of neuromuscular control in soccer players is characterized by limb asymmetries, which increases the risk of injury, especially in young players who return to play without fully recovering. This is associated with overuse injuries and increased workloads. Certain positions on the sports field have a higher risk of damage to certain anatomical areas, and important factors such as lack of training can make them more susceptible to injury (1).

According to the International Federation of Associated Football (FIFA) programs have been developed to reduce injuries, being necessary to evaluate the usefulness of neuromuscular training through the use of FIFA 11+ program, playing an important role in the reduction of injuries, which has been designed by consensus of experts and contains running exercises, strength and balance exercises, helping to improve the position of the knee, however, its real role is subject to analysis (2). Serious injuries can be effectively reduced with an injury prevention protocol in players with good results (3).

Sports injuries continue to increase and are 87% more frequent in the lower extremities (IRR: 1.87; 95% CI: 1.29−2.74) mainly in soccer and volleyball players who have a history of concussion in the last 12 months, compared to those who have not had this history, this demonstrates a direct association between neurological injuries such as concussion and other types of limb injuries (4). Athletes who suffer a concussion consequently have prolonged or slow response times, a situation that is more significant in the female group, requiring a longer neurological recovery time than clinical recovery, placing it as a group at risk of suffering new injuries (5).

Soccer is the most widely practiced sport worldwide, and the increase in injuries is understandable given its unpredictable nature, making neuromuscular control a highly relevant topic. Plyometric training involves exercises such as explosive jumping and sprinting, which have been shown to evaluate ground contact time (GCT) in milliseconds and increase muscle strength and power. This produces positive effects on speed, agility, and coordination when performing exercises that range from eccentric contractions, such as stretching, to concentric contractions, such as shortening. The average age of injury is 18.29 years. Players with a history of trauma are most frequently affected by ligament injuries (42.9%), muscle injuries (21.4%), bone injuries (14.3%), and tendon injuries (7.1%) (6).

Physical education is a subject based on physical movement. This discipline allows for the comprehensive development of a person's physical, emotional, and cognitive abilities, with the goal of improving the quality of participation. The effectiveness of trauma prevention programs with incorporated exercises reduces susceptibility to hamstring injuries; for every 1,000 h of training, injuries are reduced by 57% (7).

There are few investigated risks following a concussion in sports. Neurological changes occur in the first 14 days, such as cognitive and sleep disturbances that do not improve even with complete medical rest. Furthermore, the ideal recovery time is unknown, which represents a risk for the athlete (8).

Sport alone has shown a positive effect on the heart, for example, in hypertensive premenopausal women randomly assigned to soccer training and control group, 15 weeks of training increased left ventricular mass and improved diastolic function indices with great cardiovascular effect, so much so that soccer increased the left ventricular mass index by 10% and increased the diameter of the left ventricular septum revealing a close relationship between training and the cardiovascular system (9). Soccer also improves diseases such as type 1 diabetes with practice for 12 weeks, combined with dietary intervention, which provided an improvement in glycemia in adolescent children with type 1 diabetes (10).

FIFA 11+ training improves performance in changing direction and limb asymmetries, compared to integrative neuromuscular training. Neuromuscular training clearly improves skills in changing direction during sprinting, reducing limb asymmetries that occur with leg changes in some soccer players (11).

Neuromuscular control and cardiovascular conditioning are essential for improving endurance in soccer. Some matches last more than 120 min, and training improves the player's internal homeostasis, highlighting its importance in improving physiological aspects such as red blood cell count, lipid metabolism, and aerobic capacity (12).

Injuries represent a limitation for soccer players worldwide, as in many cases they are associated with various factors, among which the lack of adequate training stands out (13).

In youth populations, injuries can begin early, affecting the development of soccer players. A systematic review of 27 articles with data from 1427 participants reported positive effects of skills improvement programs on outcomes (14).

A systematic review of controlled trials evaluating FIFA +11 in 7,939 male soccer players with 695,503 h of training, the risk of injury was reduced by 48% (15). The following risk factors for injury in female soccer players have been identified: higher game load, history of injuries, overuse injuries at an early age and anatomical factors (16).

It is important to adapt the intensity and duration of the exercises to the level of each player, data from a meta-analysis of retrospective studies in male soccer players evaluated soccer and its effect on health (*n* = 13,065; mortality rate = 0.55; 0.46−0.67; *p* < 0.001) presenting a lower rate of neurological problems in relation to the general population (17).

Excessive training frequency is associated with several symptoms that lead to overtraining, this is related to an increased risk of injury in soccer players. A study conducted on a population of 189 young soccer players aged 13 to 17 years, showed that symptoms such as fatigue, mood swings, poor performance, anorexia, susceptibility to muscle strains, arthralgia, depression and anxiety are problems to which soccer players are exposed, so it is necessary to apply a test such as routine questionnaires to evaluate overtraining such as the one granted by the Société Françoise de Medicine du Sport (18).

Overuse knee injuries are common and are characterized by cartilage wear, which causes pain and impairs the quality of life of athletes. Lack of sleep is a factor that influences the development of overtraining syndrome in soccer players. Treatment sometimes requires advanced diagnostic and therapeutic techniques such as knee arthroscopy, knee surgery with the application of techniques such as microfracture and autologous transplantation to renew the cartilage and improve symptoms. Therefore, prevention of cartilage injury is of vital importance in sports (19).

Injuries are a growing problem for soccer players, and prevention through physical training is the subject of multiple studies that highlight its importance, given the kinematics of contact in soccer.

The practice exposes players to injuries in different parts of the body such as the head, neck, chest, abdomen, and extremities. Therefore, the training proposed to prevent injuries with advances in physical education, in addition to identifying the factors that may influence the risk of injury, is a scientific circumstance in which the importance of this research lies.

### Aim

- Defines the advances in neuromuscular training and its effectiveness in preventing injuries in soccer.

## Methods

A systematic review was conducted using articles published according to the PRISMA 2020 guidelines, obtained from databases such as PubMed, MEDLINE, Embase, CENTRAL, and Web of Science. The research design was guided by the following research question: What is the prevention, performance, and rehabilitation strategies for reducing injuries in soccer? The study population consisted of randomized controlled trials that included modifiable risk factors, advanced assessment, and training programs to prevent injuries in soccer players (20, 21).

### Eligibility criteria

The eligible sport was soccer, and the participants were either professional or amateur soccer players. The study included randomized controlled trials that incorporated interventions such as sports medicine, neuromuscular training, and conditioning, with a population comprised of professional or amateur soccer players. The entire inclusion, extraction, and synthesis process was conducted collaboratively by the authors to prevent manipulation or bias in the research. Studies with randomly selected individuals or populations were used, ensuring equal chances of selection. The interventions consisted of injury prevention, performance, and rehabilitation strategies in sports medicine. The comparator was a control group that did not receive the intervention. The required follow-up period was a minimum of one month. The primary outcome was injury prevention, and the secondary outcome included training, conditioning, rehabilitation, and physical education programs focused on soccer.

### Selection criteria

- **Participants:**
  ○ Professional and amateur soccer players.
  ○ Without gender or age restrictions.
- **Interventions**
  ○ Sports medicine
  ○ Neuromuscular training
  ○ Physical conditioning
- **Types of Studies:**
  ○ Randomized clinical trials.

### Research approach

- **Sport analyzed:** soccer.
- **Common objective in these studies:**
  ○ Reduce the risk of injuries (such as anterior cruciate ligament or ankle injuries).
  ○ Improve physical performance through structured preventative programs.
  ○ Sports medicine and conditioning

### Inclusion criteria

Randomized clinical trials concerning multicomponent neuromuscular warm-up programs to reduce injuries.
Randomized clinical trials on preventive strategies used for joint injuries or disorders in soccer players.
Randomized clinical trials related to injury prevention with strength, balance and proprioception training in soccer.
Randomized clinical trials on performance and rehabilitation strategies to prevent injuries in soccer.
Randomized clinical trials on biomechanical risk factors that may affect the practice of soccer.
Randomized clinical trials regarding educational sessions, biofeedback focused on the management and prevention of injuries.

### Exclusion criteria

Non-randomized studies
Studies on sports other than soccer
Studies unrelated to sports medicine should be excluded
Studies with unavailable results
Ongoing studies with no published data

Any study that is not about soccer should be excluded.

### Sources of information

Published articles on the usefulness of physical training in the prevention of injuries in soccer were reviewed using information from databases such as MEDLINE, Embase, CENTRAL and Web of Science.

### Search strategy

The specific search terms were neuromuscular training, warm-up, balance, proprioception, strength, FIFA 11+, knee control, Nordic hamstring training, and injury prevention.

Controlled vocabulary used English descriptors (MeSH); soccer, training, sports injuries, athletic injuries, physical activity-related injuries.

Free text terms; neuromuscular training soccer preventing injuries, Advances in neuromuscular training and its effectiveness in preventing injuries in soccer, neuromuscular training and its effectiveness in preventing injuries in soccer.

Boolean operators; “neuromuscular training soccer” AND effectiveness AND “injury prevention” AND soccer, “neuromuscular training” AND “preventing injuries” AND soccer.

Truncation; neuromuscular training *, soccer*, injuries*.

Design filters; randomized clinical trials were used to find rigorous experimental and interventional evidence.

Language limitations; research published in English.

Date limits; The search was conducted between January 2025 and final search date July 2026, including studies published over a 10-year period. For field restrictions, only articles from the last 10 years were used due to the evolution of soccer in terms of physical preparation, training methods with technology, technological advances, match load, current regulations and updated injury care protocols.

These terms were used to connect the searches in the consulted databases to link physical education and the different types of strategies used for injury prevention in soccer.

### Study selection process

The articles that responded to the objective and met the inclusion criteria were reviewed and selected jointly by all the authors of the research.

### Data extraction process

For data extraction, manual stratified randomization was used based on the study date, study type, and intervention. To ensure transparency, the extracted results were analyzed in working groups to avoid selection bias.

### List of data

The data and materials, including the sample design, instruments, hardware, software used, protocols, questionnaire, and instructions, are available from the corresponding author. Primary outcomes included injury prevention, recovery, and reduction with some form of fitness improvement training. Secondary outcomes included training load, injury incidence, injury rate, and injury risk reduction.

Participant characteristics or the primary variable for which data were collected were for professional or amateur soccer players who underwent physical training and neuromuscular control as part of their discipline in clinical studies.

### Assessing the risk of bias of individual studies

Figure 1 shows the results of the ROB2 risk of bias assessment for a specific outcome. The assessment under the intention-to-treat or per-protocol criteria resulted in a low or intermediate risk.

**Figure 1:**
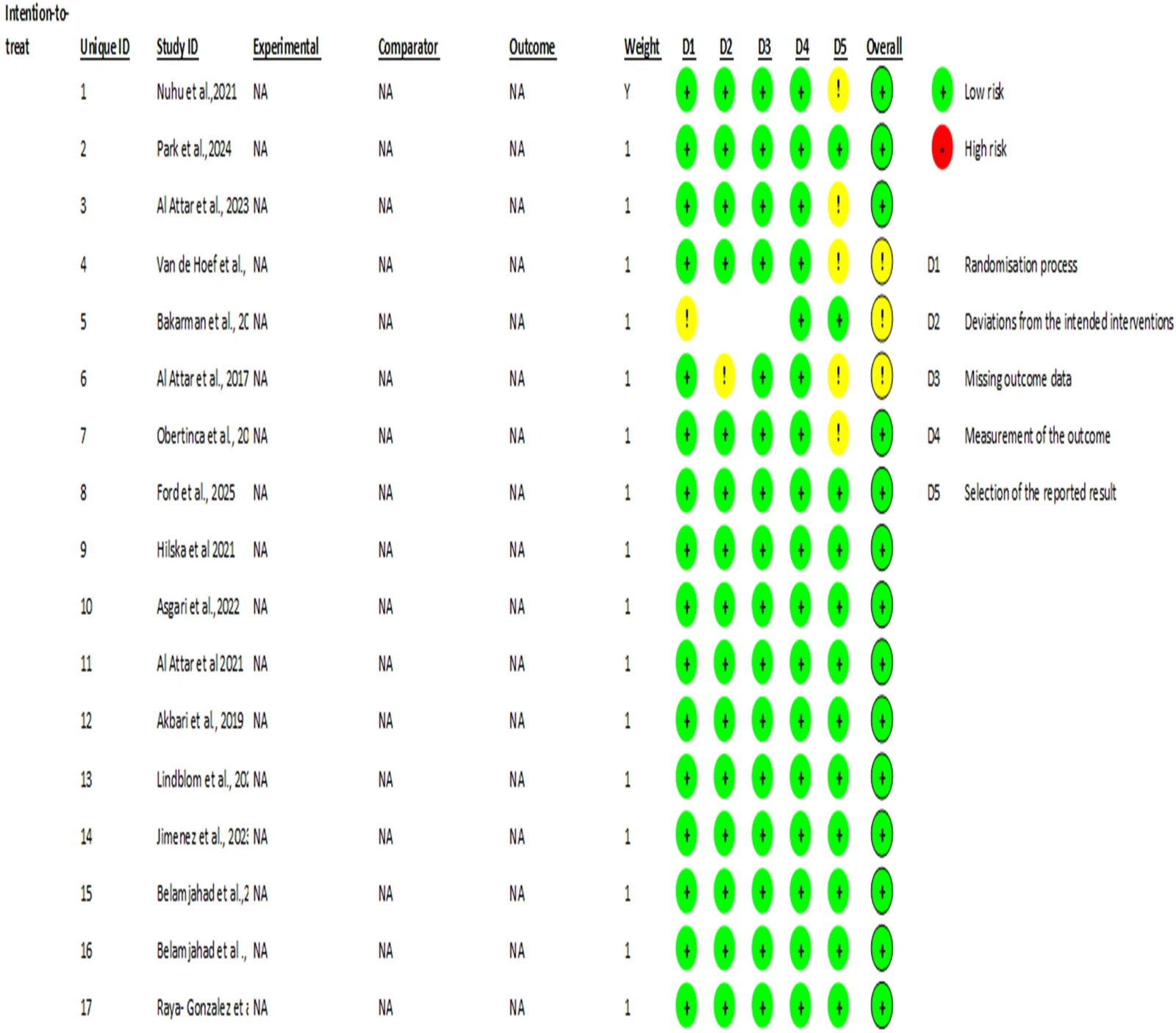
The bias assessment described in general, standardized tools such as cochrane RoB2 were used.

### Measures of effect

To measure the effect, the following were used: risk ratio (RR), odds ratio (OR), hazard ratio (HR), incidence rate ratio (IRR), relative risk (RR), confidence interval (CI), incidence ratio (IRR), absolute risk (AR), and *p*-value. The interventions consisted of advancements in physical training, neuromuscular training, and their impact on reducing injuries in soccer. A comparison was made between soccer players undergoing physical training research and those receiving regular injury-reduction training. These variables were used to estimate the protective effect, precision, and probability of the intervention.

The risk and frequency measures used, for which results were sought according to availability in the analyzed studies, were:

Risk Ratio (RR)/Relative Risk (RR): Compares the risk of injury in the advanced training group vs. the regular group.
Odds ratio (OR): Estimates the likelihood of an injury occurring in one intervention compared to another.
Instantaneous Hazard Ratio (HR): to measure the speed or rate of injuries over time between groups.
Incidence rate ratio (IRR)/IRR: to compare the number of new injuries per exposure time between the two types of training.
Absolute Risk (AR): demonstrates the actual risk or direct probability of suffering an injury in a specific group.

The measures of precision and validity that were included in the variables to assess whether the effect was significant or reliable.

Confidence interval (CI): with a 95% certainty level.

*P*-value: to measure whether the result is statistically significant or is a coincidence, if the value is < 0.05 with statistical value or >0.05 without statistical value.

### Synthesis methods

The studies selected for inclusion were synthesized using descriptive analysis and Excel result tables, as well as graphs, through a methodological filtering strategy. For the results, randomized clinical trials were considered to improve methodological rigor.

The types of training and their effect on injuries, and the participants included in Table 1, were used.

**Table 1:** Strategies for injury prevention with neuromuscular conditioning and training in soccer.

Author	Population	Intervention and training load	Results	Evaluating the bias of the evidence
Al Attar et al., 2023 (15)	780 soccer players between 7 and 13 years old were randomly assigned to an experimental group (n:391) or control group (n:389).	The FIFA 11+ Kids training program functions as a warm-up and control group for typical soccer sessions for players aged 7 to 13, twice a week for 6 months.	A 57% reduction in injuries was observed in the training group, with 43 injuries during exposure (0.85 injuries per 1,000 h). The control group had 86 injuries (2.01 injuries per 1,000 h), representing a significant reduction in injuries with the intervention (*P* = 0.001) in the knee, lower leg, and ankle compared to the control group.	Attrition bias: only 740 players completed the study, 14 dropped out in the FIFA 11+ kids training group and 26 dropped out in the control group.
Nuhu et al., 2021 (37)	(n:309) male soccer players randomly assigned to the intervention group versus (n:317) in the control group	FIFA 11+ soccer players training program for adult players. The exercises were performed at least 3 times per week for 7 consecutive months.	The intervention group had 52% of the injuries and the control group 63% (OR: 0.7; 95% CI: 0.5–0.9), showing a statistically significant reduction in total injuries (*p* < 0.001). In the intervention group, the incidence of injuries was 2.6 (95% CI: 2.2–3.0) injuries/1,000 hours of exposure, and 4.0 (95% CI: 3.5–4.5) injuries/1,000 h of exposure in the control group.	Dropout bias, program compliance was only 77%
van de Hoef et al., 2019 (39)	(n:588) male soccer players (*n* = 305) randomly assigned to the intervention group and (*n* = 283) to the control group performed the usual training	Nordic Hamstring Jumping Exercise (EII) Program in Adult Amateur Players to Prevent Hamstring Injuries, 12 Weeks of 30-Minute Jumping Exercises	With no significant difference, individualized management did not reduce the incidence or severity of hamstring injuries (*p* > 0.48) (OR = 0.89; 95% CI: 0.46–1.75). The incidence of injuries was 1.12/1,000 h in the intervention group and 1.39/1,000 h in the control group.	Churn bias in 32% of players.
Al Attar et al., 2017 (41)	Male soccer players aged 14 to 35 years were randomly assigned to an experimental group (*n* = 160 players) and a control group (*n* = 184 players) for 6 months	FIFA 11+ exercise program during pre-training for 20 min plus post-training exercises of 10 min 3 times per week, compared to the control group that performed the program only before training for 20 min	The additional post-FIFA 11+ exercise program significantly reduced the incidence of injuries by 72% (*p* = 0.001) and the baseline incidence of injuries (*p* = 0.003) (0.73 injuries/1,000 h of exposure), compared to the control group (2.6 injuries/1,000 h of exposure). The probability of sustaining an injury in the control group was significantly higher than in the experimental group [OR = 2.47 (95% CI: 1.37 to 4.46)].	Attrition bias, experimental group only 144 players completed the study and only 136 players in the control group completed the training
Obërtinca et al., 2024 (42)	(n:638) soccer players assigned to the FUNBALL program in the under-15, under-17 and under-19 categories, and (n:615) players in the control group.	The FUNBALL training program at least twice a week, compared to regular training	319 injuries with differences between groups, 132 injuries in the FUNBALL group and 187 injuries in the control group (P:0.002). With a protective effect for FUNBALL training [incidence rate ratio (IRR) 0.69, 95% CI 0.55 to 0.87].	Dropout bias: 72.2% program completion, in the FUNBALL group only 524 players completed the program and only 503 players in the control group
Ford et al., 2025 (43)	(n:150) female soccer players randomly assigned to three arms: 1. Simulated active control group with biofeedback (N:50), 2. Biofeedback group focused on knees (n:50) and 3. Biofeedback training group focused on hips (n:50).	10-minute biofeedback program with 90-minute sessions including a 9–10-minute active warm-up and 3 separate 27–30-minute sessions of each of the following: endurance training, technical/plyometric training, and strength training.	No anterior cruciate ligament (ACL) injuries were reported in any of the intervention groups. A statistically significant reduction in knee adductor moment (KAM) was observed in all 3 research groups (*p* < 0.05).	Attrition bias in all three groups; some players did not complete the study; (n: 44), (n: 48), (n: 47)
Hilska et al., 2021 (44)	1,409 soccer players in total, 684 assigned to the intervention and 740 to the control with regular training	Compliance with a neuromuscular warm-up program for injury prevention in youth soccer, neuromuscular warm-up sessions for 20 weeks, with an average of 1.7 training sessions per week	The incidence of acute injuries was 4.77 per 1,000 h of exposure in the group with high adherence to the equipment and 5.48 in the control group, with no statistical differences between the two groups (IRR 0.69, 95% CI: 0.49 to 0.97).	91% adherence, Attrition bias, 618 players completed the study out of 676 players, and 8 of the 684 did not receive the intervention.
Asgari et al., 2022 (45)	(n:90) soccer players between 16 and 19 years old randomly assigned to two intervention groups (F11+; *n* = 29, M11+; *n* = 31) and one control group (*n* = 30)	FIFA 11+ and a modified warm-up program 3 times a week for injury prevention for a period of 4 weeks	Lower limb injuries for M11+, F11+ and control group were 2.00, 5.79 and 7.55 per 1,000 h of training, with M11+ having the lowest incidence (P: 0.02)	N/A
Al Attar et al., 2021 (46)	(n:765) soccer goalkeepers randomly assigned to the experimental group (*n* = 379) or the control group (*n* = 386).	The experimental group performed the FIFA 11 + S program for 6 months compared to regular training for shoulder injury prevention.	The FIFA 11+ S group experienced 0.62 injuries per 1,000 h of exposure, while the control group experienced 1.94 injuries per 1,000 h. The total number of injuries occurring in the experimental and control groups showed a significant difference (*χ*^2^ = 40.14; *p* < 0.001). FIFA 11 + S reduced the total number of upper limb injuries by 68% [IRR = 0.32 (95% CI, 0.27–0.34)].	Attrition bias: 39 goalkeepers dropped out of the study, 80% in the FIFA 11 + S group versus 73% in the control group, according to the percentage of program completion.
Akbari et al., 2019 (47)	24 soccer players under the age of 19 were randomly assigned, 12 participants to training with FIFA 11+ compared to 12 players assigned to routine training	FIFA 11+ program 3 times per week for 8 weeks assessed by jump and landing test	A statistically significant difference was found, improving the landing pattern in participants at risk of anterior cruciate ligament (ACL) injury with the FIFA 11 + program compared to the control group (*P* = 0.031); no group experienced injuries.	N/A
Lindblom et al., 2023 (48)	(n:479) soccer players aged between 14, and 46 years were randomly assigned to two groups: Group 1, an extended knee control group compared with (n:304) players, versus Group 2; (n:175) players in an adductor strength program with exercises at three levels of difficulty	Extended Knee Control Program, two scheduled training sessions per week for 7 months	The extended knee control training program resulted in a 26% lower incidence of injuries compared to the control group (*p* < 0.001, incidence ratio 0.71, 95% CI 0.52 to 0.98). The extended knee control program included a running warm-up and six core exercises focused on muscle strength and neuromuscular control with 10 variations. The focused adductor program did not reduce groin injuries.	There was a decline bias in the participation of some of the participants, but finally 322 players completed the study.
Jiménez et al., 2023 (50)	26 professional soccer players aged 25.2 ± 3.1 years, randomly assigned to the experimental group (*n* = 16) or control group (*n* = 10)	Multicomponent training program (ProSAS) and its relationship with joint injuries	The training group had a lower incidence of joint injuries compared to the control group (*p* < 0.05).	N/A
Belamjahad et al., 2025 (51)	40 young soccer players randomized to two teams: a neuromuscular training (NMT) group (*n* = 20) and a regular training team group (*n* = 20).	Neuromuscular training applied during Ramadan for 4 weeks on physical fitness and injury prevention in youth soccer players	The injury rate was significantly lower in the neuromuscular training group (8.00/1,000 h) compared to traditional training (13.33/1,000 h) (*p* = 0.049)	N/A
Belamjahad et al., 2024 (52)	Twenty-four female soccer players, aged 17.0 ± 1.3 years, from a professional soccer club participated in this study. The players were randomly assigned to either the NMT (*n* = 12) or ET (*n* = 12) groups.	Preseason neuromuscular training program versus a resistance-dominated program on fitness and injury prevention in soccer players, weekly training sessions over a six-week period	The injury rate throughout the season was significantly lower in NMT 30% injuries in the season (5.1/1,000 h of exposure) compared to traditional training where there were 70% injuries throughout the season (11.8/1,000 h of exposure) (*p* = 0.014), incidence rate ratio of 0.43 (95% CI, 0.26–0.70).	N/A
Raya-González et al., 2021 (53)	61 U-19 soccer players were randomly assigned to a control group (n:34) or experimental group with intervention (n:27) players	Training based on Nordic hamstring and sprint exercises, over a period of 14 weeks	The incidence of injuries was not different between the two groups, although the injury burden (*p* < 0.001) favored the experimental training group: 3.82 vs. 27.87 days of absence due to injury exposure.	49 players completed the study, dropouts in both groups, only (CG; *n* = 26) (GE; *n* = 23) completed the study, participants with less than 80% compliance were excluded from the analysis.

Randomized clinical trials investigating the impact of training on injury prevention. The studies found that neuromuscular training significantly reduced injuries compared to the control group.

To visually tabulate the results of the individual studies, PICO was used and its synthesis in an Excel table.

To reduce the statistical heterogeneity of the studies, confidence intervals and statistical significance in injuries in soccer players were primarily evaluated.

### Assessment of publication bias

A critical analysis of the evidence was performed using only randomized controlled trials to eliminate randomization bias. Using the ROB2 program, trial bias was assessed as intermediate or low (see Figure 1).

### Evaluating the certainty of the evidence

The certainty of the evidence was assessed using the GRADE (Grading of Recommendations Assessment, Development and Evaluation) methodology to evaluate the certainty and strength of the recommendations.

The review findings come from both amateur and professional soccer players who practiced some type of training, warm-up, or conditioning strategy. To improve the certainty of the evidence, the research focused solely on randomized controlled trials. The populations studied included high-level evidence clinical trials conducted on children, adolescents, women, and men who were professional and amateur soccer players, with and without a history of injuries. These trials reached statistical significance for or against injury prevention through physical training.

## Results

### Selection of studies

For the development of the research, 14 randomized clinical trials that assessed the effect of training on injury prevention were identified and selected for analysis (Table 1). The studies obtained from the search were manually selected in a stratified manner among all authors according to the results to issue clear recommendations of a high level of evidence and were expressed in the flow diagram that represents the direction of the research (Figure 2) (22).

**Figure 2:**
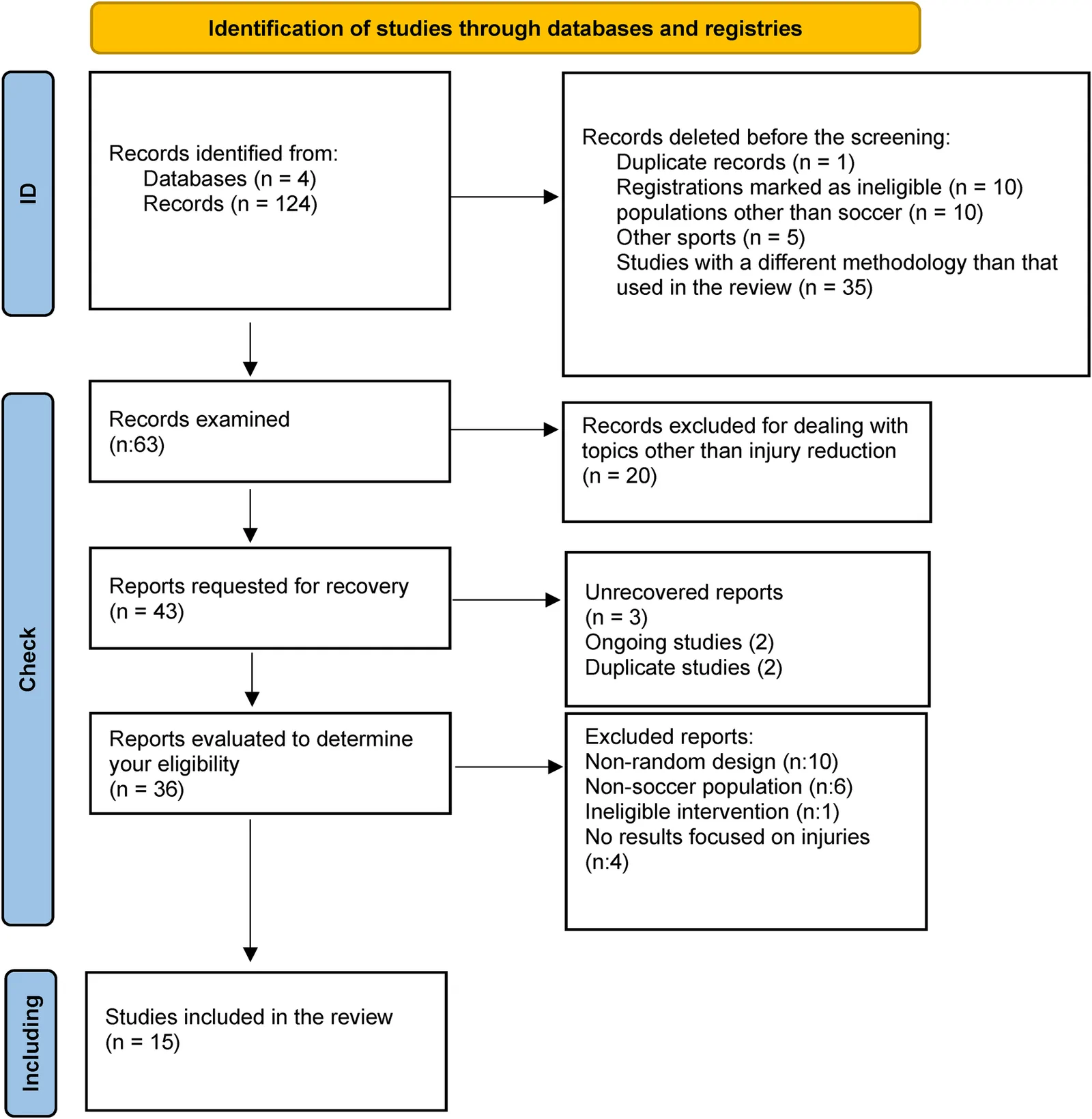
PRISMA 2020 flowchart. Research flowchart according to the PRISMA 2020 statement. 17 randomized clinical trials were obtained because of the final screening. All clinical trials that did not meet the inclusion criteria were excluded.

Studies that apparently met the inclusion criteria, but with a focus on other interventions that were not injury prevention, were excluded (23–36, 49); other strategies that included conditioning for soccer were taken into consideration (Figure 2, and Table 2).

**Table 2:** Strategies focused on improving skills, performance and conditioning in soccer.

Author	Population	Intervention and training load	Results	Evaluating the bias of the evidence
Arede., et al. 2021 (11)	(n:30) male soccer players with an average age of 11.2 ± 0.7 years, equally randomized to comprehensive neuromuscular training or control with usual training to improve performance and changes in asymmetries	Comprehensive neuromuscular training versus regular training	Significant improvements in the change of direction test (*p* < 0.01), significant decrease in sprint time from 0 to 20 (*p* < 0.05)	N/A
Park et al., 2024 (38)	(n:51) Elite male adolescent soccer players with functional ankle instability recruited into strength, balance and control groups (balance *n* = 17; strength, *n* = 17; and control, *n* = 17, respectively)	Strength and balance training to reduce functional ankle instability and kinesiophobia assessed using the Tampa scale	Strength and balance training reduced functional instability of the ankle joint and kinesiophobia (*P* < 0.001)	N/A
Bakarman et al., 2022 (40)	(n:502) amateur soccer players, (n:246) randomly assigned to the intervention group and (n:256) to the control group	Assessment of changes in knowledge of injuries and their impact on skills	Significantly higher scores than the control group in knowledge of all aspects related to injuries (*p* < 0.0001). Physical education regarding injury mechanisms, prevention, and treatment significantly improves knowledge and skills.	N/A

Randomized clinical trials on strategies to improve skills, knowledge, and landing patterns in soccer.

There are other variables such as the application of FIFA 11+ neuromuscular training to reduce asymmetries during leg changes or changes in speed rhythm during the training run that provide improvements in leg asymmetries and changes of direction during the run, although the study did not assess the effect on injuries, it only evidently improved skills (11).

The intervention program for injury risk factors such as functional instability consisted of: first, strengthening the ankle in three directions, second, single-leg heel raises, and third, balance training for 6 weeks in players >14 years old with a history of functional ankle instability, who were assessed using the Tampa scale to measure the fear of re-injury, in addition to quantifying and evaluating the degree of kinesiophobia of the participants, which showed significant improvement by decreasing kinesiophobia, instability, and improving performance after the program. For soccer players with a history of ankle injuries, individualized training significantly reduced ankle instability (38).

Educational intervention in amateur soccer players with a median age of 22 years improves educational knowledge and performance of soccer players, as well as their technical skills, although it does not directly reduce injuries. An educational session taught Saudi soccer players cognitively about the role of physical training and its relationship to injuries and disseminated educational content in the research group for 90 min, which included teaching the causes of injuries, injury mechanisms, types of injuries, symptoms, prevention measures and treatment. It did not decrease the incidence of injuries, only affected the knowledge of these in the sports medicine training group (40).

### Characteristics of the studies

The GRADE methodology was used to classify the quality of the scientific evidence from the selected studies. This research used a high level of evidence, including randomized trials.

### Risk of bias of individual studies

An individual analysis was performed for each study selected in Table 1 with the respective risk of bias dependent on participant dropouts.

### Results of individual studies

The results of each study included in the selection were summarized individually in Table 1, and are described according to population, intervention, comparison, and outcomes (PICO).

### Results of the synthesis

The probability of injury occurring in children < 14 years was lower in the FIFA 11+ exercise group applied for 6 months. The FIFA 11+ kids consisted of 7 exercises with 5 levels of difficulty performed as a warm-up before training sessions, which significantly reduced injuries in relation to the control group (15).

FIFA 11+ exercises as an intervention applied in the warm-up showed a protective effect for players who performed the training for 7 in relation to the control group that did not perform FIFA 11+ (37).

Although the Nordic hamstring exercise had a protective effect on injuries by reducing them, the probability of the event was lower in the group exposed to the program in relation to the unexposed control group, the prevention of hamstring injuries in adult male soccer players did not reach statistically significant value (39).

The probability of suffering an injury with exercises in male players with the FIFA 11+ program applied for 6 months before and after training was significantly lower in the intervention group compared to the control group that only applied warm-up exercises before training (41).

The implementation of the FUNBALL program after the usual warm-up of soccer players between 15 and 19 years old twice a week resulted in the exposed group obtaining injury occurrence rates 31% lower compared to the non-exposed group with statistical significance (42).

Biofeedback was used in female soccer players with a mean age of 13.3 ± 2.1 years, employing sensors to measure involuntary bodily functions. This biofeedback indicated the amount of load or force exerted on the inner part of the knee. Real-time biofeedback training reduced knee adduction moment, improving performance in landing and jumping tests. No anterior cruciate ligament injuries were recorded in any of the three groups that underwent biofeedback. One limitation of the study was the lack of a control group without intervention; the effect of this control group was not measured (43).

The results demonstrate a protective effect was observed with the neuromuscular warm-up program in male and female soccer players with a mean age of 12 years who performed 2 to 3 weekly sessions for 20 min for 20 weeks as an intervention. An Incidence Rate Ratio (IRR) of 0.77 (95% CI: 0.61 to 0.96) was obtained, indicating a 23% decrease in the event rate in the exposed group compared to the control group. Non-contact lower extremity injuries were reduced in the teams with high adherence. The study found no statistically significant difference in the incidence of acute injuries, nor in the prevalence of injuries. The warm-up had no statistically significant effect on overuse injuries of the lower extremities. In the high-adherence group, the incidence was 12.5% (95% CI: 11.6 to 13.5) compared to 11.3% (95% CI: 10.7 to 11.9) in the control group (44).

Soccer players aged 16 to 19 were compared across two training programs: FIFA 11+ and M11+. The latter is a modified version of the M11+ program that includes: first, core exercises (prone, supine, and dynamic plank), balance exercises, and iliopsoas muscle strengthening. The second phase consists of straight-line running, controlled contact drills, hip inward and outward tilts, dynamic hamstring stretching, and rotational and cutting movements. The third phase comprises Nordic hamstring exercises, single-leg balance with a heel-to-toe movement cycle, ball throwing, and squats. The final part, agility and plyometric exercises, including drop jumps and countermovement jumps, injury prevention, and performance enhancements, practiced three times a week with warm-ups before training sessions for four months, reduced lower limb injuries. The M 11+ resulted in 2.00 injuries per 1,000 hours of training vs. 5.79 injuries per 1,000 hours for FIFA 11+ and 7.55 injuries per 1,000 hours with regular training. The magnitude of the effect on injuries was not evaluated; a protective effect was only observed on skill, time, and speed (45).

Another variant of FIFA 11+ is FIFA 11+ S (or FIFA 11+ Shoulder) which is an official variant of the system for preventing upper limb injuries to the shoulder, elbow, wrist and fingers in goalkeepers in a statistically significant way (46).

Regarding the FIFA 11+ program as a conditioning exercise practiced 3 times a week for 8 weeks in elite soccer players under 19 years of age (mean ±: age = 16.79 ± 1.18 years) with risk factors for cruciate ligament injury, it improves the ability in terms of landing mechanics in relation to the control group, although no cruciate ligament injuries were observed in either group (47).

Extended Knee Control as a program to prevent hamstring, knee and ankle injuries, knee injuries in the research group decreased in relation to the control and there was no effect of extended adductor training on injuries in this area (48).

The ProSAS training with a mean age of 25.2 ± 3.1 years, achieved a statistically significant reduction in injuries with 8 weeks of training (50).

The overall injury rate in high-performance male players aged 17-18 years was significantly lower in neuromuscular training (8.00 injuries per 1,000 hours of exposure) compared to traditional training (13.33 injuries per 1,000 hours of exposure) (*p* = 0.049) (51).

The injury rate was significantly lower in the neuromuscular training group (5.1/1,000 h of exposure) compared to the endurance training group using running and sprinting, fartlek and high-intensity interval training (11.8/1,000 h of exposure) (*p* = 0.014) (52).

Studies based on Nordic exercises in the context of soccer were accompanied by an injury burden in favor of the experimental group (27.87 days of absence in the control group vs. 3.82 days of absence in the intervention group per 1,000 hours of exposure, rate ratio = 7.30, 95% CI: 3.34-15.99) (53).

### Publication biases

A high percentage of attrition bias was observed, with high rates of participant dropout, both selectively and systematically, due to the long-term nature of the studies, which reduced precision because the data did not represent the original group that was initially randomized. The ROB2 program was used to assess bias, which individually revealed intermediate and low risk in most studies.

The statistical heterogeneity dependent on the different statistical measures evaluated was reduced by using the *P* value as the main variable to measure significance, using a cutoff point of *P* < 0.05 or *P* > 0.05 as significant or insignificant, improving the synthesis with results for and against training in relation to injuries.

### Certainty of the evidence

The certainty of the evidence was assessed using the GRADE system. The final analysis used 15 randomized controlled clinical trials with a high level of evidence to evaluate the types of training, physical conditioning, and their direct effect on injuries in soccer.

A lower incidence of injuries was observed in the training group compared to the control group, which did not perform regular training not focused on a multi-skills strategy plan. Some studies revealed a protective effect of training; the *p*-value was influenced by a high dropout rate and a smaller sample size than in the intervention group (37). The incidence of injuries was lower in the experimental group with neuromuscular training compared to the control group, although the control group trained fewer hours. Dropouts occurred in both groups, introducing attrition bias (15). Hamstring injuries are common in both groups of soccer players, with an incidence of 1.12 per 1,000 hours in the jump training group and 1.39 per 1,000 hours compared to the regular control group, although the results did not reach statistical significance. When analyzing surgical procedures, a protective effect of jump training was observed (39).

A 10 min FIFA 11+ exercise after training was accompanied by a significant reduction in injuries; at the start of the study there was a smaller population in the training group compared to the control group (41).

Dropout rates were high in both groups. In the intention-to-treat analysis, a significant difference was observed between the two groups, with fewer injuries in the FUNBALL training group (42).

The implementation of feedback using technology such as biofeedback for 10 min was accompanied by good results due to the absence of injuries in all study groups (43). An important factor in neuromuscular training is adherence to training; the study showed that high adherence is associated with a lower injury rate (44).

The modified program called M11+, which includes specific exercises, yielded better results, significantly reducing injuries compared to FIFA 11+ (45). Although FIFA 11+ youth training was associated with a notable reduction in the percentage of injuries, the analysis of the results did not account for players who dropped out of the study after randomization, so the results may have been influenced by the likelihood of dropping out (46). Nevertheless, FIFA 11+ reduces the incidence of injuries with statistical significance, supporting the program's effectiveness in reducing anterior cruciate ligament injuries (47).

In a population where knee injuries were evaluated, training was associated with a lower incidence of injuries compared to the control group (48).

ProSAS training was associated with fewer joint injuries compared to the control group (50). The neuromuscular training group achieved statistical significance in injury reduction, with no dropouts observed during the 4 weeks of training (51, 52). A longer time off after injury was observed, but no statistical significance was observed in the incidence of injuries; both groups had an equal number of injuries (53).

In 76.47% of the studies analyzed, a reduction in injuries or improvements in conditioning, adherence, jump mechanics, and joint stability were achieved with a statistically significant value (*P* < 0.0134). Studies in which no improvements in injury rate or conditioning were observed represented 23.52%, therefore, future research should focus on providing additional data on this topic.

The research provides precise data on the types of training that did or did not demonstrate a protective effect, which is repeated with neuromuscular training, obtaining statistical significance when applied as a warm-up, conditioning before and after soccer. With programs such as FIFA 11+ compared to the usual warm-up, FIFA 11+ Kids showed a significant reduction when applied before and after practice, presenting a greater protective effect than when applied only before practice. This indicates that the primary outcome improved with this type of training. FIFA 11+ S is a variant of the FIFA 11+ warm-up protocol, developed for the prevention of shoulder injuries in soccer goalkeepers also showed a significant protective result.

The extended FIFA 11+ Kids program stands out as the most used training program among young people; other additional programs also obtained good results, FIFA 11 + S, M11+, ProSAS and FUNBALL, Extended Knee Control, although they need more studies to replicate the primary result.

## Discussion

Neuromuscular training, the application of technology, and teaching can significantly reduce injuries despite limitations in studies (54).

Other evidence, such as a systematic review of non-randomized studies in the context of training, revealed a 10-fold reduction in injuries, reporting 36 injuries per 1,000 hours of exposure, reduced to 3.7 injuries per 1,000 hours of training (54).

A meta-analysis of randomized clinical trials investigated the best soccer training to reduce anterior cruciate ligament injuries, and the highest scoring were FIFA 11+ and Knee Control, observing a significant impact of training (55). Other injuries are common in populations such as women, where training is highly relevant in prevention, due to the evidence of current results (56).

Evidence shows poor program adherence and limited research on the causes of dropouts and their relationship to injuries. Data are based on heterogeneous populations to integrate results on a large scale; However, more high-quality research in specific populations is needed.

Future research should be directed towards improving current programs with strategies based on improvements in physical exercise and policies of follow-up and intervention aimed at injuries (57).

To assess the real effect of training, specific injury prevention programs are needed, focused on the most common areas of the group of soccer players who do not perform training focused on a clear objective (58).

## Conclusions

The review demonstrates the importance of conditioning studies with neuromuscular training and the programs currently used, including: FIFA 11+, FIFA 11+ Kids, FIFA 11+ S, FUNBALL, ProSAS, M11+, FUNBALL, and Extended Knee Control before or after training, which prevent injuries in professional and amateur soccer players, with a protective effect and statistically significant effect in participants who developed this practice for 2 to 7 continuous months for a minimum of 10 min between 2 and 3 times per week compared to the control group that did not perform focused training.

Other types of conditioning included improvements in physical performance through preventative programs with no statistically significant effect on injury rate, for example: extended adductor program, neuromuscular training alone, Nordic training in injury load, biofeedback, adherence to training and landing mechanics improve neuromuscular control and favorably influence conditioning.

The review acknowledges interventions in sports medicine that can influence the mechanism of injury, for example, interruption of conditioning, kinesiophobia as a factor in ankle injury recovery with specialized ankle strength and balance training focused on improving skills, as well as implementation of technology in soccer to recognize patterns associated with injuries, they also did not have a significant effect on injury prevention.

## References

[1] Mandorino M Figueiredo AJ Gjaka M Tessitore A. Injury incidence and risk factors in youth soccer players: a systematic literature review. Part II: intrinsic and extrinsic risk factors. Biol Sport. (2023) 40(1):27–49. 10.5114/biolsport.2023.109962 36636190 PMC9806737

[2] Alhazmi M Alhazmi E Alghamdi WA Zalah M Uddin S Rizvi MR. Effectiveness of FIFA injury prevention programs in reducing ankle injuries among football players: a systematic review. PeerJ. (2025) 13:e18910. 10.7717/peerj.18910 40084177 PMC11905912

[3] Moncer R Ghanmi M Chebil D Bhiri S Belhadj Youssef I Kacem A. Only severe injuries are effectively reduced by muscle injury prevention protocols in soccer players: a systematic review. F1000Res. (2024) 13:695. 10.12688/f1000research.148925.2 40177023 PMC11963548

[4] Biese KM Kliethermes SA Watson AM McGuine TA Lang PJ Bell DR. Musculoskeletal injuries and their association with prior concussion history: a prospective study of high school volleyball and soccer players. Am J Sports Med. (2021) 49(6):1634–41. 10.1177/0363546521997116 33719583

[5] Wilkerson GB Wynn KR Dill PW Acocello S Carlson LM Hogg J. Concussion history and virtual reality metrics predict core or lower extremity injury occurrence among high school athletes. Facing Sports Life. (2024) 6:1374772. 10.3389/fspor.2024.1374772 PMC11004318 38600904

[6] Monsalve Vélez F Betancur Henao S Buriticá Ochoa D Gómez Urán D Mira Peña AI Tabares Castaño WV. Effect of plyometric training on neuromuscular control and lower limb flexibility in the Cosdecol men’s soccer team. Rev Dígito Act Deportar. (2021) 7(1):1–11. 10.31910/rdafd.v7.n1.2021.1529

[7] Al Attar WSA Husain MA. Effectiveness of injury prevention programs with core strengthening exercises in reducing the incidence of hamstring injuries among soccer players: a systematic review and meta-analysis. Sports Health. (2023) 15(6):805–13. 10.1177/19417381231170815 37139743 PMC10606958

[8] Leddy JJ Burma JS Toomey CM Hayden A Davis GA Babl FE. Rest and exercise early after sports-related concussion: a systematic review and meta-analysis. Br J Sports Med. (2023) 57(12):762–70. 10.1136/bjsports-2022-106676 37316185

[9] Sjúrðarson T Kyhl K Nordsborg NB Kollslíð R Andersen LJ Krustrup P. Fifteen weeks of soccer training increases left ventricular mass and improves indices of left ventricular diastolic function in previously sedentary, mildly hypertensive, middle-aged women. Eur J Appl Physiol. (2024) 124(5):1621–9. 10.1007/s00421-023-05399-7 PMC11055800 38177568

[10] Mohammed MHH Al-Qahtani MHH Takken T. Effects of 12 weeks of calorie-controlled recreational soccer on glycemia and cardiovascular health in adolescent boys with type 1 diabetes. Pediatr Diabetes. (2021) 22(4):625–37. 10.1111/pedi.13203.AND 33745203

[11] Arede J Fernandes J Moran J Leite N Romero-Rodriguez D Madruga-Parera M. Effects of an integrative neuromuscular training protocol vs. FIFA 11+ on sprint performance, change of direction and interlimb asymmetries in young soccer players. Int J Sports Sci Coach. (2021) 17(1):54–62. 10.1177/17479541211011438

[12] Kurtoğlu A Konar N Akçınar F Alotaibi MH Elkholi SM. The response of the amputee athlete’s heart to chronic core exercise: a study on hematological and biochemical parameters, and N-terminal brain natriuretic peptide. Medicine (Kaunas). (2024) 60(5):784. 10.3390/medicine60050784 PMC11123260 38792967

[13] Stergiou M Calvo AL Forelli F. Effectiveness of neuromuscular training in preventing lower extremity soccer injuries: a systematic review and meta-analysis. J Clin Med. (2025) 14(5):1714. 10.3390/jcm14051714 40095804 PMC11899896

[14] Jiménez-García JA Miller MB DeMont RG. Effects of multicomponent injury prevention programs on fundamental movement skills in children and adolescents: a systematic review with meta-analysis. Am J Health Promotion. (2023) 37(5):705–19. 10.1177/08901171221146434 PMC10580683 36527377

[15] Al Attar WSA Bizzini M Alzahrani H Alarifi S Ghulam H Alyami M. The FIFA 11+ kids injury prevention program reduces injury rates among male child soccer players: a clustered randomized controlled trial. Sports Health. (2023) 15(3):397–409. 10.1177/19417381221109224 35903029 PMC10170224

[16] Burbano Carrera JX Avalos Tipán MA. Injury prevention in women’s football: impact of the FIFA 11+ program. JGH. (2024) 1(3):313–3. 10.59282/jgh1(3)313-333

[17] Morales JS Valenzuela PL Saco-Ledo G Castillo-García A Carabias CS McCrory P. Risk of mortality from neurodegenerative disease in sports associated with repetitive head impacts: preliminary findings from a systematic review and meta-analysis. Sports Med. (2022) 52(4):835–46. 10.1007/s40279-021-01580-0 34674184

[18] Rodrigues F Monteiro D Ferraz R Branquinho L Forte P. The association between training frequency, overtraining symptoms, and injuries in young soccer players. Int J Environ Res Public Health. (2023) 20(8):5466. 10.3390/ijerph20085466 37107750 PMC10138529

[19] Bonilla Obando MH Gonzalez Cetina NF Cárdenas Ojeda SP López Velandia DP. Relationship between sleep quality and early manifestations of physical overtraining syndrome in Colombian soccer players. Challenges. (2024) 58:1134–40. 10.47197/retos.v58.101031

[20] Samala AD Rawas S Criollo-C S Bojic L Prasetya F Ranuharja F. Emerging technologies for global education: a comprehensive exploration of trends, innovations, challenges, and future horizons. SN Compute Sci. (2024) 5:1175. 10.1007/s42979-024-03538-1

[21] Samala AD Mhlanga D Bojic L Howard N-J Coelho DP. Blockchain technology in education: opportunities, challenges, and beyond. Int J Interact Mob Technol. (2024) 18(01):20–42. 10.3991/ijim.v18i01.46307

[22] Page MJ McKenzie JE Bossuyt PM Boutron I Hoffmann TC Mulrow CD. The 2020 PRISMA statement: updated guidance for reporting systematic reviews. Br Med J. (2021) 372:N71. 10.1136/bmj.n71 PMC8005924 33782057

[23] Kritikos S Papanikolaou K Draganidis D Poulios A Georgakouli K Tsimeas P. Effect of whey protein versus soy protein supplementation on recovery kinetics after sprint endurance training in competitive male soccer players: a randomized controlled trial. J Int Soc Sports Nutr. (2021) 18(1):23. 10.1186/s12970-021-00420-w 33726784 PMC7968192

[24] Emirzeoğlu M Ülger Ö. The acute effects of cognition-based neuromuscular training and game-based training on dynamic balance and sprint performance of healthy young soccer players: a randomized controlled trial. Games Health J. (2021) 10(2):121–9. 10.1089/g4h.2020.0051 33170049

[25] Hammami A Selmi W Mahmoudi A Negra Y Chaouachi A Behm DG. Effects of neuromuscular training versus stretching performed during warm-up on measures of physical fitness and mental well-being in highly trained pubertal male soccer players. PLoS One. (2025) 20(2):e0318318. 10.1371/journal.pone.0318318 39982921 PMC11844867

[26] Vasileiou SS Asimakidis ND Dalamitros AA Manou V. Effects of an 8-week in-season explosive power training program on neuromuscular performance and lower limb asymmetries in young male soccer players. J Strength Cond Res. (2024) 38(12):2121–8. 10.1519/JSC.00000000000004917 39178053

[27] Vera-Assaoka T Ramirez-Campillo R Alvarez C Garcia-Pinillos F Moran J Gentil P. Maturational effects on fitness adaptations to drop jump plyometric training in male youth soccer players. J Strength Cond Res. (2020) 34(10):2760–8. 10.1519/JSC.00000000000003151 32986391

[28] Westbrook AE Taylor JB Nguyen AD Paterno MV Ford KR. Effects of maturation on knee biomechanics during cutting and landing in youth female soccer players. PLoS One. (2020) 15(5):E0233701. 10.1371/journal.pone.0233701 32453805 PMC7250454

[29] Menezes GB Alexandre DRO Pinto JCBL Assis TVL Faigenbaum AD Mortatti AL. Effects of integrative neuromuscular training on motor performance in prepubertal soccer players. J Strength Cond Res. (2022) 36(6):1667–74. 10.1519/JSC.00000000000003666 32796417

[30] Chnini Z Salem A Trabelsi K Ammar A Souissi N Chtourou H. Light-load jump squat and plyometric training improve jumping, running, change of direction, and balance performance of male (U-19) soccer players: a randomized controlled trial. J Sports Med Phys Fitness. (2024) 64(8):719–27. 10.23736/S0022-4707.24.15368-6 38841726

[31] Hasan S Kandasamy G Alyahya D Alonazi A Jamal A Unnikrishnan R. Effect of resisted sprint and plyometric training on lower extremity functional performance in male collegiate soccer players: a randomized control trial. Int J Environ Res Public Health. (2021) 18(13):6702. 10.3390/ijerph18136702 34206346 PMC8295600

[32] Aloui G Souhail H Hayes LD Bouhafs EG Chelly MS Schwesig R. Effects of combined plyometric and short sprint training on the athletic performance of male U-19 soccer players. Front Psychol. (2021) 12:714016. 10.3389/fpsyg.2021.714016 34603139 PMC8481369

[33] Aloui G Hermassi S Bartels T Hayes LD Bouhafs EG Chelly MS. Combined plyometric and short sprint training in U-15 male soccer players: effects on measures of jumping, speed, change of direction, repeated sprinting, and balance. Frontal Physiology. (2022) 13:757663. 10.3389/fphys.2022.757663 PMC8895237 35250606

[34] Padrón-Cabo A Alonso-Calvete A Radzimiński Ł Rey E Lorenzo-Martínez M. Acute effects of foam rolling versus foam roller vibration on blood flow parameters in professional soccer players: a randomized crossover design. J Strength Cond Res. (2024) 38(11):1885–90. 10.1519/JSC.00000000000004896 39178061

[35] Schwebel DC Long DL McClure LA. Youth soccer field injuries: do additional referees reduce risk? A randomized crossover trial. J Pediatr Psychol. (2020) 45(7):759–66. 10.1093/jpepsy/jsaa050 32651582 PMC7381189

[36] Silvers-Granelli H Silverman R Bizzini M Thorborg K Brophy RH. The 11+ injury prevention program decreases the rate of hamstring strain injuries in male collegiate soccer players. Br J Sports Med. (2024) 58(13):701–8. 10.1136/bjsports-2023-107323 38599679

[37] Nuhu A Jelsma J Dunleavy K Burgess T. Effect of the FIFA 11+ soccer specific warm up program on the incidence of injuries: a cluster-randomised controlled trial. PLoS One. (2021) 16(5):e0251839. 10.1371/journal.pone.0251839 34029321 PMC8143390

[38] Park HS Oh JK Kim JY Yoon JH. The effect of strength and balance training on kinesiophobia, ankle instability, function, and performance in elite adolescent soccer players with functional ankle instability: a prospective cluster randomized controlled trial. J Sports Sci Med. (2024) 23(1):593–602. 10.52082/jssm.2024.593 39228771 PMC11366847

[39] Van De Hoef PA Brink MS Huisstede BMA Van Smeden M De Vries N Goedhart EA. Does a bounding exercise program prevent hamstring injuries in adult male soccer players? - A cluster-RCT. Scand J Med Sci Sports. (2019) 29(4):515–23. 10.1111/sms.13353 30536639 PMC6850185

[40] Bakarman MA Tashkandi M Mohammed NS. An intervention plan for preventing and handling amateur soccer players’ injuries. Niger J Clin Pract. (2022) 25(11):1816–22. 10.4103/njcp.njcp_237_22 36412288

[41] Al Attar WSA Soomro N Pappas E Sinclair PJ Sanders RH. Adding a post-training FIFA 11+ exercise program to the pre-training FIFA 11+ injury prevention program reduces injury rates among male amateur soccer players: a cluster-randomised trial. J Physiother. (2017) 63(4):235–42. 10.1016/j.jphys.2017.08.004 28939307

[42] Obërtinca R Meha R Hoxha I Shabani B Meyer T Aus der Fünten K. Efficacy of a new injury prevention program (FUNBALL) in young male football (soccer) players: a cluster-randomised controlled trial. Br J Sports Med. (2024) 58(10):548–55. 10.1136/bjsports-2023-107388 PMC11103335 38499320

[43] Ford KR Taylor JB Westbrook AE Paterno MV Huang B Nguyen AD. Outcomes of a randomized controlled trial of neuromuscular training with real-time biofeedback in young female athletes. BMC Musculoskelet Disord. (2025) 26(1):366. 10.1186/s12891-025-08647-3 40234828 PMC11998317

[44] Hilska M Leppänen M Vasankari T Aaltonen S Raitanen J Räisänen AM. Adherence to an injury prevention warm-up program in children’s soccer-A secondary analysis of a randomized controlled trial. Int J Environ Res Public Health. (2021) 18(24):13134. 10.3390/ijerph182413134 34948744 PMC8701740

[45] Asgari M Alizadeh MH Shahrbanian S Nolte K Jaitner T. Effects of the FIFA11+ and a modified warm-up program on injury prevention and performance improvement among youth male football players. PLoS One. (2022) 17(10):e0275545. 10.1371/journal.pone.0275545 36264894 PMC9584367

[46] Al Attar WSA Faude O Bizzini M Alarifi S Alzahrani H Almalki RS. The FIFA 11+ shoulder injury prevention program was effective in reducing upper extremity injuries among soccer goalkeepers: a randomized controlled trial. Am J Sports Med. (2021) 49(9):2293–300. 10.1177/03635465211021828 34138672

[47] Akbari H Sahebozamani M Daneshjoo A Amiri-Khorasani M Shimokochi Y. Effect of the FIFA 11+ on landing patterns and baseline movement errors in elite male youth soccer players. J Sport Rehabil. (2020) 29(6):730–7. 10.1123/jsr.2018-0374 31629326

[48] Lindblom H Sonesson S Torvaldsson K Waldén M Hägglund M. Extended knee control program lowers weekly hamstring, knee and ankle injury prevalence compared with an adductor strength program or self-selected injury prevention exercises in adolescent and adult amateur football players: a two-armed cluster-randomized trial with an additional comparison arm. Br J Sports Med. (2023) 57(2):83–90. 10.1136/bjsports-2022-105890 36316115 PMC9872240

[49] Peek K Versteegh T Veith S Whalan M Edwards S McKay M. Injury reduction programs containing neuromuscular neck exercises and the incidence of football-related head and neck injuries. J Athl Train. (2023) 58(6):519–27. 10.4085/1062-6050-0340.22 36645836 PMC10496449

[50] Jiménez-Rubio S Raya-González J Lobo-Triviño D García-Calvo T. The impact of a multicomponent training program on fitness and joint injuries in Spanish professional soccer players. J Sports Sci. (2023) 41(21):1934–43. 10.1080/02640414.2024.2307780 38258651

[51] Belamjahad A Tourny C Hackney AC Laziri F Saeidi A El Hachimi O. Effects of neuromuscular training applied during Ramadan on physical fitness and injury prevention in highly trained male youth soccer players. Sports Med Open. (2025) 11(1):34. 10.1186/s40798-025-00831-y 40192962 PMC11977061

[52] Belamjahad A Tourny C Jebabli N Clark CCT Laher I Hackney AC. Effects of a preseason neuromuscular training program versus a resistance-dominated program on physical fitness and injury prevention in female soccer players. Sports Med Open. (2025) 10:76. 10.1186/s40798-024-00731-7 PMC11208342 38922502

[53] Raya-González J Torres Martin L Beato M Rodríguez-Fernández A Sánchez-Sánchez J. The effects of Nordic hamstring and sprint-based exercise training on fitness measures and hamstring injury prevention in U-19 male soccer players. Sports Med Res. (2021) 31(5):588–603. 10.1080/15438627.2021.2010206 34841995

[54] López-Valenciano A Ruiz-Pérez I Garcia-Gómez A Vera-Garcia FJ De Ste Croix M Myer GD. Epidemiology of injuries in professional football: a systematic review and meta-analysis. Br J Sports Med. (2020) 54(12):711–8. 10.1136/bjsports-2018-099577 PMC9929604 31171515

[55] Magaña-Ramírez M Gallardo-Gómez D Álvarez-Barbosa F Corral-Pernía JA. What exercise program is the most appropriate to mitigate anterior cruciate ligament injury risk in football (soccer) players? A systematic review and network meta-analysis. J Sci Med Sport. (2024) 27(4):234–42. 10.1016/j.jsams.2024.02.001 38395699

[56] Crossley KM Patterson BE Culvenor AG Bruder AM Mosler AB Mentiplay BF. Making football safer for women: a systematic review and meta-analysis of injury prevention programs in 11,773 female football (soccer) players. Br J Sports Med. (2020) 54(18):1089–98. 10.1136/bjsports-2019-101587 32253193 PMC7497572

[57] Olivares-Jabalera J Fílter-Ruger A Dos’Santos T Afonso J Della Villa F Morente-Sánchez J. Exercise-based training strategies to reduce the incidence or mitigate the risk factors of anterior cruciate ligament injury in adult football (soccer) players: a systematic review. Int J Environ Res Public Health. (2021) 18(24):13351. 10.3390/ijerph182413351 34948963 PMC8704173

[58] Pfirrmann D Herbst M Ingelfinger P Simon P Tug S. Analysis of injury incidences in male professional adult and elite youth soccer players: a systematic review. J Athl Train. (2016) 51(5):410–24. 10.4085/1062-6050-51.6.03 27244125 PMC5013706

